# Lateralization of auditory steady state response (ASSR) deficits in first-episode schizophrenia – Effects of attention and associations with auditory hallucination severity

**DOI:** 10.1016/j.jpsychires.2025.11.004

**Published:** 2025-11-07

**Authors:** Brian A. Coffman, Xi Ren, Julia Longenecker, Natasha Torrence, Vanessa Fishel, Dylan Seebold, Yiming Wang, Mark Curtis, Lauren Fowler, Hayley Rhorer, Jack Kavanagh, Francisco Lopez-Caballero, Alfredo Sklar, Dean F. Salisbury

**Affiliations:** Clinical Neurophysiology Research Laboratory, Western Psychiatric Hospital of UPMC, Department of Psychiatry, University of Pittsburgh School of Medicine, Pittsburgh, PA, USA

## Abstract

Psychotic disorders, characterized by perceptual abnormalities and cognitive decline, affect millions of people. Auditory hallucinations (AH), or the perception of non-existent sounds, are particularly taxing. While AH is often viewed as a perceptual disorder, patient distress is more closely related to cognitive factors. Research suggests that dysfunctional attentional control of auditory systems may contribute to AH development, and auditory cognitive control may be a key factor in the link between AH and functional decline. This study used magnetoencephalography (MEG) to investigate attentional control of ASSR in first-episode psychosis (FEP) within left and right primary auditory cortex (A1) at initial clinical contact and 4–12 months later. We investigated the relationships among ASSR deficits and attention-mediated sensory gain in 40 FEP and 40 matched healthy comparison subjects (HC). We measured ASSR to click trains with attention directed toward or away from the auditory stimulus at both timepoints. Results indicated that FEP patients showed reduced attentional modulation of ASSR, particularly in the right A1, and increased ASSR during passive listening in left A1, correlating with AH severity. ASSR measurements were reliable, with persistent group differences despite symptom reduction. These findings highlight the role of selective attention deficits in psychosis and suggest ASSR as a potential biomarker for temporal lobe dysfunction. Early identification of ASSR deficits could enable targeted treatments for individuals at highest risk of developing a chronic disorder.

## 1. Introduction

Millions of people suffer from symptoms of psychotic disorders such as schizophrenia, which are characterized by perceptual abnormalities and severe cognitive decline (Cloutier et al., 2016). The perception of sounds or voices that do not exist in the physical environment, or auditory hallucination (AH), is among the most distressing of these symptoms, interfering significantly with daily life. Although AH is a widely considered a perceptual disorder, patient distress from AH is more strongly linked to cognitive factors, such as perceived controllability or conviction regarding the biological source of AH, than physical perceptual characteristics, such as perceived loudness or proximity (Birchwood and Chadwick, 1997). Research suggests that dysfunctional attentional control of auditory systems may contribute to AH development, and auditory cognitive control may be a key factor in the link between AH and functional decline (Hugdahl, 2009; Hugdahl et al., 2008; Waters et al., 2012). Accordingly, the field has shifted to investigating dysfunctional attentional control of auditory systems as a potential precipitating factor in the development of AH.

The strength of primary auditory evoked response in superior temporal lobe is sensitive to the effects of attention beginning approximately 50 ms after stimulus onset (Bidet-Caulet et al., 2007; Hansen et al., 1983; Woldorff et al., 1993). We and others have shown that this response is not only reduced in psychosis, but that attentional modulation of this response is also blunted significantly, even at first episode (Coffman et al., 2023, 2024; Curtis et al., 2023; Gudlowski et al., 2009; Juckel et al., 2003; Ren et al., 2021; Sklar et al., 2023a, 2023b, 2024). Additionally, early auditory processing is sensitive to the effects of attention, as indexed by attention-related changes in amplitudes of N100 auditory evoked potentials (Coch et al., 2005; Hansen et al., 1983; Määttä et al., 2005), and we have shown that these effects are reduced in first-episode psychosis (FEP) (Coffman et al., 2023; Curtis et al., 2023). One of these basic auditory processes, the auditory steady state response (ASSR) reflects the “resonant” local circuit dynamics of auditory cortex, and phase-locks strongly to acoustic stimuli at 40 Hz (Brenner et al., 2009). Reduced ASSR power is considered a stable index of auditory circuit dysfunction associated with schizophrenia and other related psychiatric disorders (Roach et al., 2019; Tada et al., 2020; Tan et al., 2015). Furthermore, the ability to suppress ASSR during periods of diverted attention may be directly related to AH severity, particularly for cognitive factors associated with AH (Coffman et al., 2023).

The majority of ASSR research has utilized electroencephalographic (EEG) measurement to investigate effects of psychosis. The first EEG study examining ASSR and AH in schizophrenia reported that increased left hemisphere ASSR was related to greater AH severity (Spencer et al., 2009), and a subsequent study showed that interhemispheric synchrony of the ASSR was also related to more severe AH (Mulert et al., 2011); however, this method lacks high spatial resolution to make precise and substantive inferences about the cortical sources of dysfunction in these individuals. Cognitive control deficits in schizophrenia, however, are predominantly identified in right hemisphere (Zhao et al., 2024), and auditory cognitive control is highly right lateralized in healthy volunteers (Petit et al., 2007). It is not known whether deficits in attentional control of ASSR within primary auditory cortex are lateralized, or whether the relationship between these deficits and AH is related to hemispheric asymmetry of such attentional control (Coffman et al., 2022).

Here, we utilized magnetoencephalography (MEG) to investigate attentional control of ASSR in FEP within left and right primary auditory cortex (A1) at first clinical contact and again 4–12 months later. We measured ASSR to click trains during passive listening and with attention to the auditory stimulus. Based on our prior findings, we hypothesized that FEP would show reduced attention modulation of ASSR and that the degree to which attention modulation was reduced would correlate with AH severity. We further predicted that this relationship would be driven by reduced suppression of ASSR during passive listening, reflecting hyperactivity of auditory sensory circuit functioning putatively due to reduced frontal inhibition. We predicted that this effect would be most prominent in left auditory cortex, given left lateralization language function and tendency for AH to be perceived as verbal expressions, and that this pathologically increased left A1 ASSR during periods of inattention would correlate with AH severity in FEP.

## 2. Methods

### 2.1. Participants

Forty FEP and 40 healthy comparison (HC) individuals participated. We successfully acquired follow-up MEG from 23 FEP and 24 HC. HC were recruited to approximately match FEP group mean age, sex, parental socioeconomic status (pSES) assessed by the Hollingshead Index (Hollingshead, 2011), and IQ assessed by the Wechsler Abbreviated Scale of Intelligence (WASI-I) (Weschler, 1999), and follow-up HC assessments were selected to match FEP on these variables post-hoc, resulting in 24 follow-up HC datasets (see Table 1 for demographics). Participants were excluded for a) history of concussion or head injury with sequelae, b) history of alcohol or drug addiction or detox in the last five years, c) presence of neurological disease or disorder, d) < 9 years education, or e) IQ < 75. All subjects had normal hearing assessed by audiometry (within 30 dB nHL and <15 dB difference between ears from 1000 to 4000 Hz). All participants completed the MATRICS Cognitive Consensus Battery (Nuechterlein et al., 2008). Participants provided voluntary informed consent and were compensated for participation. Procedures were approved by the University of Pittsburgh IRB.

All FEP participated within their first episode of psychosis and all but one had <3-months lifetime antipsychotic medication exposure. Diagnosis was based on the Structured Clinical Interview for DSM-IV-TR (SCID-IV) and consensus conference review, initially at protocol entrance and again 5–7 months after initial clinical assessment based on all available longitudinal data. Broad symptoms were rated using the Positive and Negative Symptom Scale (PANSS) (Kay et al., 1987). Symptoms were also assessed with the AH scale of the PSYRATS, which provides scores for physical, emotional, and cognitive factors of AH using 11 Likert-scale (0–4) questions (Drake et al., 2007). The delusions scale of the PSYRATS was also administered. Participants were asked to rate their responses according to symptom severity over the prior 2-week period. All interviews and tests were conducted by an expert (Masters’- or PhD-level) clinical assessor. Of the 40 FEP participants, 21 received diagnoses of Schizophrenia (paranoid, n = 10; undifferentiated, n = 11), two of Schizoaffective Disorder (bipolar type, n = 1; depressive type, n = 1), 11 received diagnoses of a primary affective disorder with psychotic features (Bipolar 1 Disorder, n = 6; Major Depressive Disorder; n = 5), and three of Psychotic Disorder NOS. FEP lost to follow-up for research purposes included two (10 %) diagnosed with Schizophrenia (paranoid, n = 1; undifferentiated, n = 1), two (100 %) with Schizoaffective Disorder, and seven (64 %) with primary affective disorders with psychotic features (Bipolar 1 Disorder, n = 3 [50 %]; Major Depressive Disorder; n = 4 [80 %]) Three FEP participants were also completely lost to follow-up (clinically as well as research) and therefore remain with the diagnosis of Schizophreniform Disorder (Provisional). Analysis without these participants did not produce different results, nor did exclusion of the 11 participants with primary affective disorder diagnosis, most of whom were lost to follow-up for research. See Table 2 for clinical characteristics.

### 2.2. Procedures

Participants were exposed to binaural auditory click stimuli created with Audacity and delivered via Presentation software (Neurobehavioral Systems, Inc.) through Etymotic 3A insert earphones. This was done in two blocks, each containing 450 stimuli. Concurrently, a silent nature video was shown. In one block, participants were instructed to focus on and count the auditory stimuli (pressing a button every 7th stimulus) while ignoring the video. In the other block, they were to ignore the auditory stimuli and watch the video. The order of the blocks was counterbalanced, with a short (3–4 min) break between blocks. Stimuli consisted of 1 ms biphasic clicks at a rate of 40 Hz (500 ms duration, with a stimulus onset asynchrony of 750–1150 ms). Participants were monitored via video to ensure they remained awake and directed their gaze towards the silent video throughout the task. Research associates were trained to engage participants in conversation to build rapport and reduce anxiety prior to and during testing. Participants were also introduced to testing environments prior to data collection, both to ensure no sources of electromagnetic noise were present (e.g. dental work, underwire bra, etc.) and to reduce anxiety during data collection. Noise sources were removed or degaussed, as appropriate.

### 2.3. ASSR measurement

MEG data were recorded in a magnetically shielded room with a 306-channel whole-head system (Elekta Neuromag), consisting of 128 triplets (1 magnetometer and two planar gradiometers) at 1000Hz digitization with a bandpass filter of 0.1–330Hz. Eye blinks and movements were recorded with bipolar leads placed above and below the left eye and lateral to the outer canthi of both eyes. Cardiac activity was recorded with bipolar ECG leads. A 3D-digitizer (ISOTRAK; Polhemus, Inc., Colchester, VT) was used to continuously record the location of four head position indicator coils. Neuromag MaxFilter software (http://imaging.mrc-cbu.cam.ac.uk/meg/Maxfilter_V2.2) was used to correct for head motion. Temporal signal space separation was used to remove electromagnetic noise originating from outside the MEG helmet (Uusitalo and Ilmoniemi, 1997). Channels and segments of data with excessive noise via visual inspection were removed with EEGLAB (Delorme and Makeig, 2004). A high-pass filter (0.5Hz; 12 dB/oct) was applied to the data, and an adaptive mixture independent component analysis was performed to remove eye-blink and ECG components. Any removed channels were then interpolated.

MEG processing was performed with Brainstorm (Tadel et al., 2011). A low-pass (59Hz) filter was applied to remove line noise and other high-frequency artifacts. Trials were then segmented from 200 ms before to 700 ms after stimulus onset, average baseline signal was subtracted, and trials that exceeded ±5 pT were rejected. MEG sensor data was registered to each participant’s structural MRI, obtained within 4 weeks of the corresponding MEG dataset. Sources were constrained to the individual’s cortical surface. The forward solution was modeled as overlapping spheres, and a noise covariance matrix was calculated from the baseline window of all trials (both blocks). Source activity was estimated using minimum norm estimation with a dipole constraint of 0.4 and depth-weighting applied. Current density values were normalized with dynamic statistical parametric maps (dSPM). For calculation of ASSR power, Morlet wavelet deconvolution was applied to each trial using 5 cycles at 1 Hz increments from 15 to 50 Hz. Baseline signal power was then subtracted at each frequency to isolate stimulus-related fluctuations in the gamma-band. ASSR power was measured as the average across 35–45 Hz and 200–400 ms post-stimulus.

The HCP-pipelines (https://github.com/Washington-University/HCPpipelines) were used for MRI processing (Glasser et al., 2013). Group average HCP-MulitModal Parcellation was applied to individuals’ cortical surface in native space for identification of the left and right auditory cortex ROIs. For a complete description of MRI data acquisition parameters and processing procedures, see Curtis et al. (2023). Auditory cortex ROIs comprised left and right A1.

### 2.4. Data analysis

Group demographics and button-press frequency were compared between groups using t-tests and chi-squared tests where appropriate. To utilize the full statistical power at baseline assessments, we used two separate repeated measures ANOVA analyses. We first compare baseline ASSR power between groups, attention conditions, and cortical region of interest (ROI; left vs right A1) with a 2 × 2 × 2 design, then assess these effects longitudinally by including a fourth within-subject factor (time: baseline or follow-up). Analysis of baseline ASSR was also repeated with a 3 × 2 × 2 design, where FEP were divided into those with (AH+; N = 18) and those without AH (AH-; N = 22). Simple effects were examined using *t*-test, between-subjects ANOVA or repeated-measures ANOVA as appropriate. Sequential multiple regression was used to assess whether passive ASSR in left A1 and active ASSR in right A1 predict AH severity. These tests were performed within AH + as well as across all FEP, where presence/absence of AH symptoms was entered into the model as a dichotomous categorical variable to account for variance in-homogeneity (Robertson et al., 1994). Bivariate Pearson correlations were used for follow-up tests assessing relationships with individual PSYRATS factor scores (physical, emotional, and cognitive factors).

## 3. Results

FEP did not differ from HC in demographic composition (e.g., age, sex, parental SES), apart from having lower education level and slightly different distribution of race (Table 2). There were no significant differences in task performance during the attend condition (*p*’s > 0.1); HC pressed the button once for every 7.9 stimuli (0.4 SD) on average, while FEP pressed the button once for every 7.8 stimuli (0.3 SD). Within FEP, AH + pressed the button every 7.7 stimuli (0.3 SD), while AH− responded to every 7.9 stimuli (0.2 SD).

**Fig. 1.**
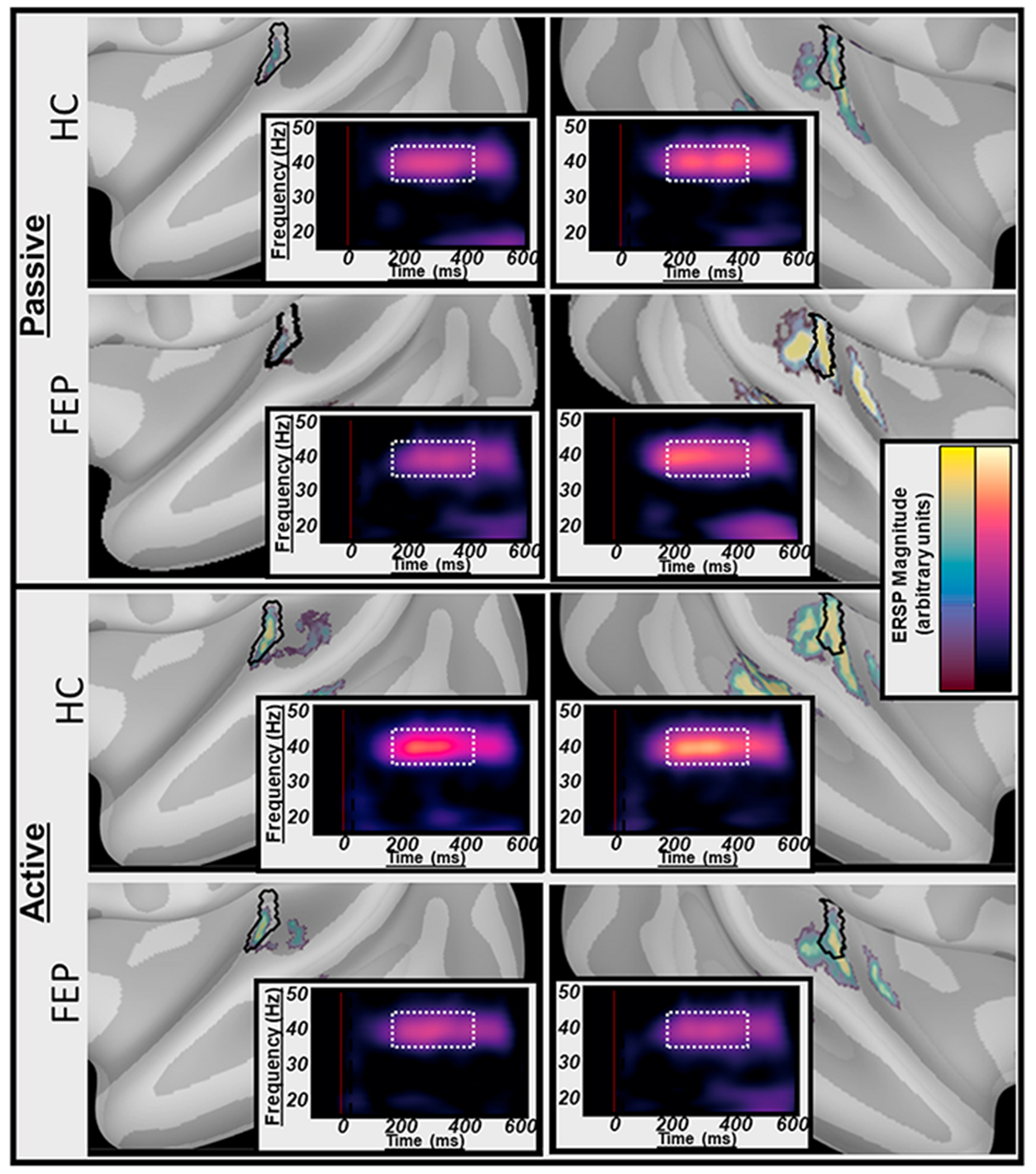
Baseline ASSR. Spectrograms and source localization of ASSR measurements at baseline in HC (N = 40) and FEP (N = 40). ASSR is depicted separately for left and right auditory cortex in left and right panels, and for passive and active listening conditions in upper and lower panels, respectively. White dashed lines in spectrograms indicate the frequency band and time window depicted in background source locations, and black solid outline in source localizations indicate the patch of cortex depicted in foreground spectrograms.

**Fig. 2.**
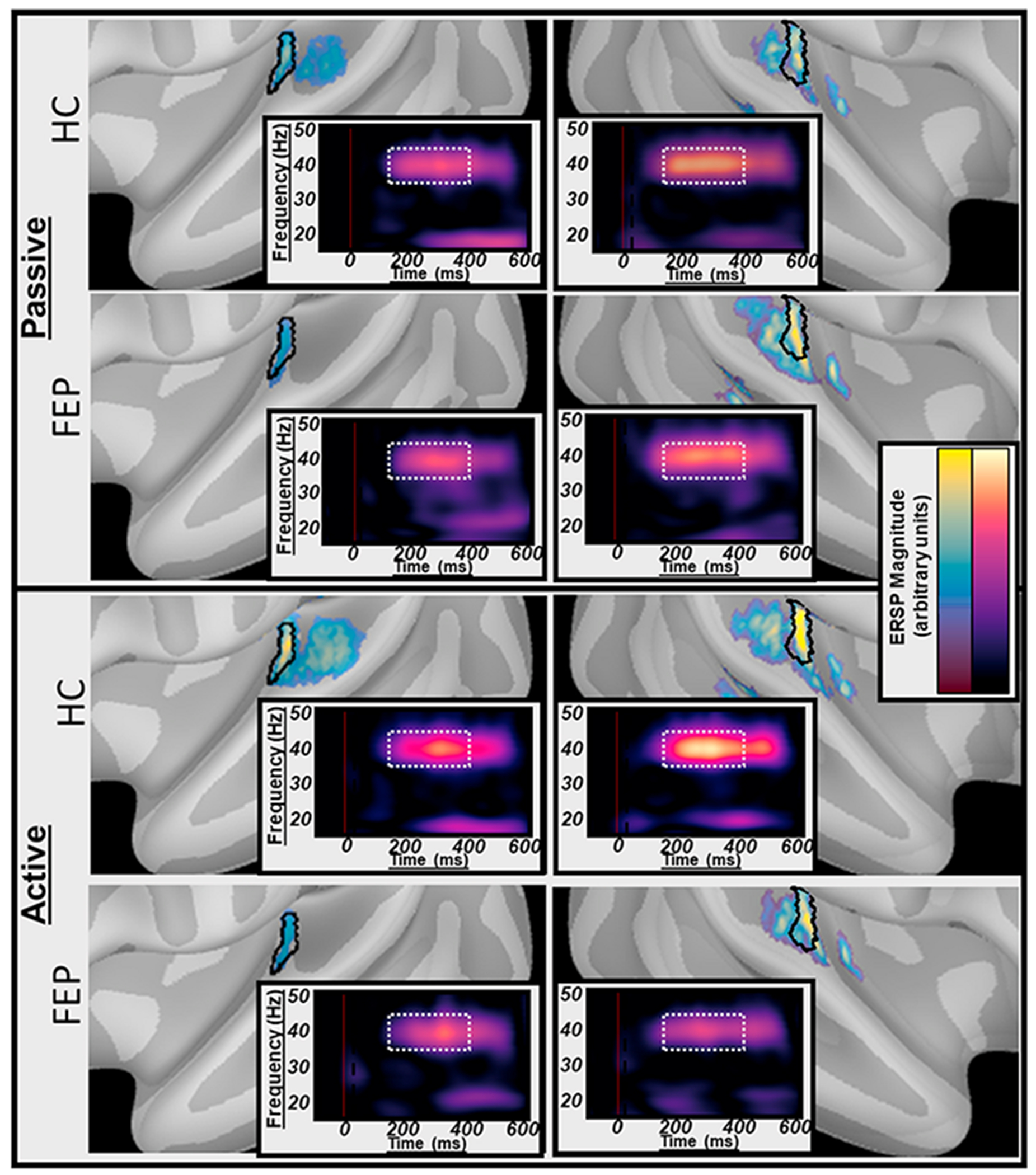
Follow-up ASSR. Spectrograms and source localization of ASSR measurements at follow-up in HC (N = 23) and FEP (N = 23). ASSR is depicted separately for left and right auditory cortex in left and right panels, and for passive and active listening conditions in upper and lower panels, respectively. White dashed lines in spectrograms indicate the frequency band and time window depicted in background source locations, and black solid outline in source localizations indicate the patch of cortex depicted in foreground spectrograms.

Although average ASSR power at baseline across stimulus conditions in both hemispheres was not different between FEP and HC, attention conditions, or left and right A1 (*p* > 0.1), ASSR group differences were identified dependent upon attention condition and cerebral hemisphere (3-way interaction (*F*(_1,78_) = 4.76; *p* = 0.032). The effect of attention on ASSR differed between FEP and HC in right A1 (group × attention interaction; *F*(_1,78_) = 9.76; *p* = 0.003) but not left A1 (p > 0.1). Within right A1, ASSR increased with attention to sounds in HC (attend = 2.2 ± 0.3 nAm^2^, ignore = 1.6 ± 0.3 nAm^2^; *p* = 0.007); but not in FEP (*p* > 0.1). There were no differences between attention conditions or groups within left A1 (*p* > 0.1). ASSR in right A1 during attention to sounds was also greater in HC than FEP (FEP Attend = 1.4 ± 0. 2 nAm^2^; *p* = 0.009), while groups were not different for passive ASSR in right A1 (FEP Ignore = 1.5 ± 0.2 nAm^2^; *p >* 0.1). More generally, there was no difference in ASSR between attention conditions or left/right A1 nor interaction between attention condition and hemisphere in FEP (*p* > 0.1). In HC, however, each of these effects was present (hemisphere: *F*(1,39) = 7.56; *p* = 0.009; attention: *F*(_1,39_) = 8.11; *p* = 0.007; interaction; *F*(_1,39_) = 4.54; *p* = 0.039), where ASSR was most prominent in right A1, and this lateralization was stronger with attention to sounds (*F*(1,39) = 9.22; *p* = 0.004) than during passive listening (*F*(_1,39_) = 3.87; *p* = 0.056). ASSR source localizations and spectrograms are shown for each group and condition in Fig. 1 (Baseline) and Fig. 2 (Follow-up).

Dividing FEP into AH+ and AH− subgroups revealed additional complexity to these results, particularly for passive ASSR in left A1. Demographic and cognitive variables were closely matched between AH+ and AH-; however, clinical measures showed expected differences between groups, where AH + had greater positive symptoms, including delusions, as well as increased general symptoms and lower medication dosage, both current and lifetime (Table 3). A three-way interaction (*F*_(2,78)_ = 4.22; *p* = 0.018), again indicated that group differences varied by attention condition and hemisphere. Furthermore, between-group differences in active ASSR again varied by hemisphere (*F*_(2,77)_ = 5.36; *p* = 0.006), where HC active ASSR (2.2 ± 0.3 nAm^2^) was greater than both AH− (1.2 ± 0.2 nAm^2^; *p* = 0.04) and AH+ (0.8 ± 0.2 nAm^2^; *p* = 0.009) within right A1 (*F*_(2,77)_ = 4.38; *p* = 0.016), while no differences in active ASSR were found between AH+ and AH− within right A1 (*p* > 0.1) and no group differences in active ASSR were identified in left A1 (*p* > 0.1). Unlike our initial model, between-group differences in passive ASSR now also varied by hemisphere (*F*_(2,77)_ = 5.46; *p* = 0.006), with group differences in passive ASSR identified left A1 (*F*_(2,77)_ = 3.30; *p* = 0.042), but not right A1 (*p* > 0.1). Passive ASSR in left A1 was greater for AH+ (1.8 ± 0.3 nAm^2^) than AH− (0.9 ± 0.2 nAm^2^; *p* = 0.032), while neither was significantly different from HC (1.3 ± 0.3 nAm^2^; *p*’*s* > 0.1).

Longitudinal assessment of these effects in a subset of participants (23 FEP and 24 HC) between 4 and 12 months after initial testing identified no changes in ASSR differences between groups, stimulus conditions, lateralization, or any interaction among these variables (4-way interaction p > 0.1). This was also the case when dividing the FEP group into those with (AH+) and those without AH (AH-), or when excluding those with a primary affective diagnosis (N = 4). Moreover, magnitude and pattern of ASSR differences between groups/conditions were largely preserved in this analysis (*F*(_1,45_) = 14.68; *p* < 0.001), with group differences restricted to ASSR in right A1 during attention to sounds (HC > FEP; *F*(_1,45_) = 9.43; *p*=0.004). Stability of ASSR measurements between timepoints was high for HC (ICC_Attend_ = 0.54; ICC_Ignore_ = 0.52) and moderate for FEP (ICC_Attend_ = 0.41; ICC_Ignore_ = 0.34), with no significant within-group differences over time (*p* > 0.1).

**Fig. 3.**
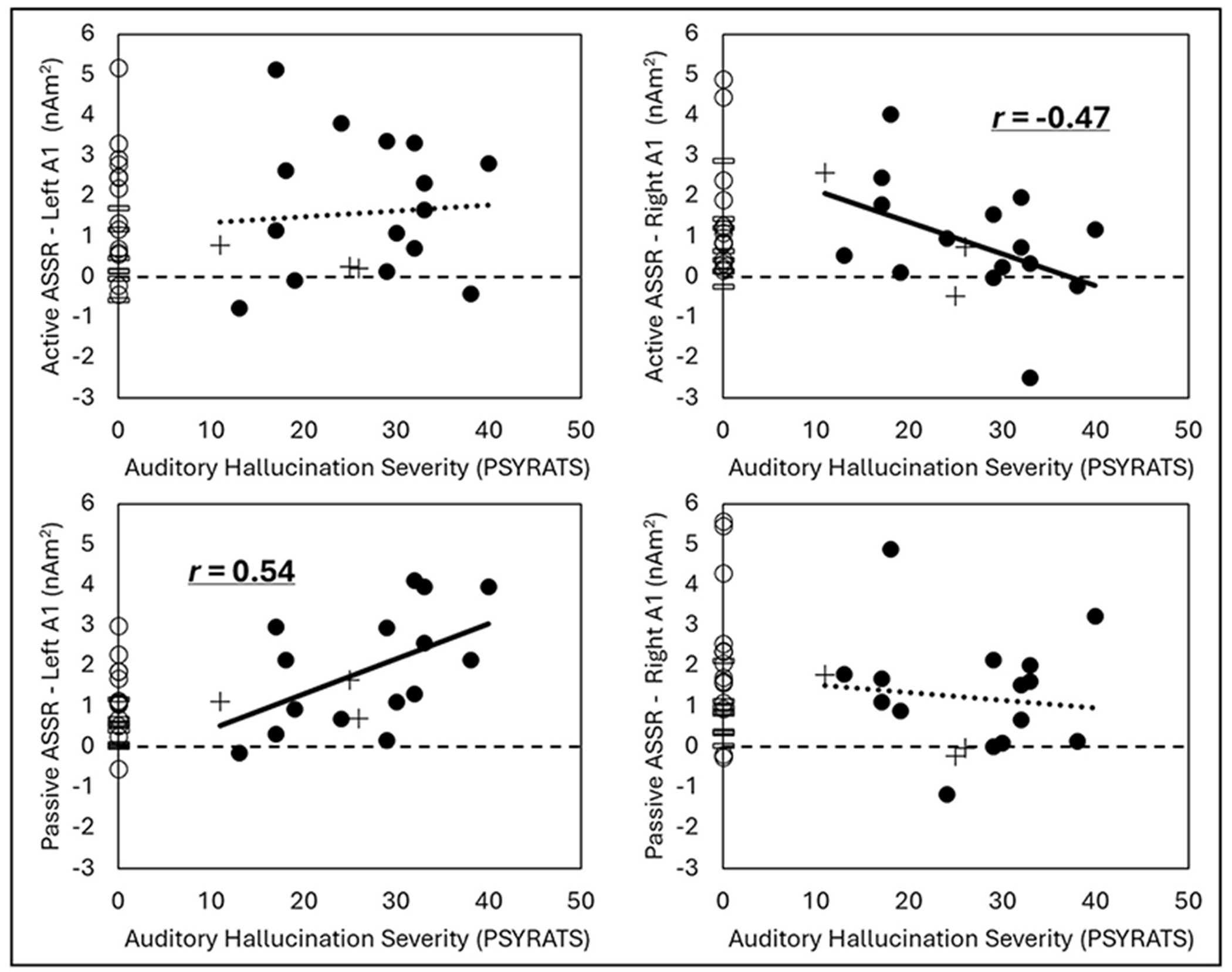
Scatterplots showing the relationship between auditory hallucination severity and ASSR during active attention to sounds (upper panels) and passive listening (lower panels). ASSR measured from left and right A1 are depicted in the left and right panels, respectively. Individuals diagnosed with affective-spectrum psychotic disorders are depicted by + symbols, while those with schizophrenia-spectrum disorders are shown as circles.

Tables 4 and 5 display the unstandardized regression coefficients (*B*), standardized regression coefficients (β), and partial correlations for sequential regression models with and without AH− participants included in the model, respectively. The regression model predicting AH severity from ASSR in left A1 during passive listening and right A1 during attention to auditory stimuli was significant, controlling for overall presence/absence of AH (*R*^2^ = 0.90, *F*(3,36) = 111.2, *p* < 0.001), where left A1 ASSR accounted for a significant proportion of the variance in AH over and above presence/absence of AH (Δ*R*^2^ = 0.03, *F* (1,37) = 9.40, *p* = 0.004) and right A1 ASSR with attention accounted for a significant proportion of AH variance over and above left A1 ASSR and AH presence/absence (Δ*R*^2^ = 0.03, *F*(1,36) = 9.70, *p* = 0.004). For every 1-SD increase in left A1 ASSR power during the ignore condition, AH severity increased by 2.7 points (*t*(39) = 3.91, *p* < 0.001), while each 1-SD increase in right A1 ASSR during the attend condition was related to a decrease of 1.7 points (*t*(39) = −3.01, *p* = 0.004). Within AH+ (N = 18), the regression model predicting AH severity from both passive ASSR in left A1 and active ASSR in right A1 was significant (*R*^2^ = 0.55, *F* (2,15) = 9.1, *p* = 0.003), where active ASSR in right A1 accounted for a significant proportion of the variance in AH over and above passive ASSR in left A1 (Δ*R*^2^ = 0.25, *F*(1,15) = 8.43, *p* = 0.011). Within AH+, there were significant bivariate correlations between left A1 ASSR during the passive condition and auditory hallucination severity as measured by PSYRATS total (Fig. 3; *r* = 0.54; *p* = 0.02) and physical factor scores (*r* = 0.53; *p* = 0.02), as well as between right A1 ASSR during the active condition and auditory hallucination severity as measured by PSYRATS total (Fig. 3; *r* = −0.47; *p* = 0.05) and cognitive factor scores (*r* = −0.49; *p* = 0.04).

## 4. Discussion

Psychosis often leads to cognitive and sensory problems, such as difficulty executing goal-directed behavior (Barch, 2005; Cohen et al., 1999) and deploying attention toward or away from sensory stimuli (Bellgrove et al., 2003; Carter et al., 1997). Here, we replicate our prior findings of reduced attentional modulation of ASSR in FEP (Coffman et al., 2022) and further show that this reduction is related to attentional modulation in right A1, but not left A1. In fact, HC also generally had a greater ASSR in the right A1 compared to left A1 and showed significant modulation of ASSR in the right but not left A1. Additionally, we find that ASSR measurements show moderate to high reliability over time in both groups, and that differences between groups persist at follow-up 4–12 months after initial measurements, despite reductions in psychosis symptoms over this period. We have shown previously that these deficits are related to auditory hallucination severity, such that failure to enhance ASSR response was related to cognitive aspects of auditory hallucination severity (e.g. the ability to ignore or control the impact of hallucinations on behavior). Conversely, failure to suppress ASSR response measured with EEG was related to greater experience of physical aspects of auditory hallucinations, such as loudness and proximity (Coffman et al., 2022). In the present study, we have further elucidated the nature of this relationship by identifying a lateralization of functional differences underlying auditory hallucination severity.

Rightward lateralization of attention effects on auditory processing has been demonstrated previously using intracranial EEG and MEG (Gomez-Ramirez et al., 2011; Müller and Weisz, 2012; Weisz et al., 2014), as well as temporal lobe lesion studies in which the excision of right, but not left auditory cortex predicted difficulties in attending to and localizing sounds (Zatorre and Penhune, 2001). Reduced ability to modulate ASSR with attention therefore aligns with cognitive control deficits in the population, further evinced by correlation between right hemisphere ASSR with attention and cognitive factors associated with auditory hallucination severity in FEP. The inability to modulate sensory responses with attention in FEP may contribute to a wide range of cognitive impairments, such as difficulties with working memory, sustained and selective attention, and verbal learning. Furthermore, reduced auditory attentional control in FEP could directly impact social cognition, as effective social interactions often depend on the ability to selectively focus on auditory stimuli. This skill is crucial for understanding speech in noisy environments (Anderson et al., 2013) or when listening to non-native-accented speech (Van Engen and Peelle, 2014). Deficits in sensory attention and vigilance have long been recognized as a core cognitive deficit in psychosis (Bleuler, 1911). Our finding of reduced ASSR modulation with attention in FEP patients supports this notion, highlighting deficits even at the level of basic auditory neurophysiology.

Although group differences were not identified within left hemisphere in this study, passive ASSR (but not active ASSR) in left A1 was related to the physical experience of hallucinations here. This factor score includes items such as loudness and perceived proximity of voices, characteristics that modulate auditory cortex responses to real/physical sounds as well. Given generally left-lateralized nature of language and semantic networks, this positive relationship between left auditory excitability at rest and physical experiences of auditory hallucinations aligns with over-excitability/deafferentation hypotheses of auditory hallucinations (Boksa, 2009; Marschall et al., 2020), as well as prior reports of positive associations between left hemisphere ASSR and auditory hallucinations (Spencer et al., 2009).

Some caveats for these findings include small sample size, diagnostic diversity of the sample, differences in racial distribution between FEP and HC groups, and potential impact of anxiety or other psychiatric comorbidities. This population is quite difficult to sample, given factors such as the time-limited nature of assessment of FEP and the need for clinical stability in assessment of the ability to consent to research. Future studies may seek to sample from multiple sites and across diverse cultural, socioeconomic, or genetic characteristics. Such a large-scale endeavor would also facilitate subgroup analyses specific to diagnostic categories, in addition to analysis across the psychosis spectrum. Additionally, inclusion of individuals with long-term/chronic illness may inform the degree to which long-term antipsychotic treatment might affect ASSR. Finally, although we strive to reduce the effects of stress during data collection here, impacts of anxiety on neurophysiological measures are undoubtedly present in all human research.

Our study illustrates the significant cognitive and sensory challenges faced by individuals with psychosis, particularly in their ability to execute goal-directed behavior and manage attention toward auditory stimuli. We replicated previous findings of reduced attentional modulation of ASSR in FEP and identified the right auditory cortex (A1) as the putative source of this attentional deficit. Our findings further elucidate the relationship between ASSR deficits and auditory hallucination severity, where the inability to enhance ASSR in right A1 is associated with cognitive aspects of hallucinations and passive ASSR in left A1 relates to the physical experience of hallucinations. The lateralization of these functional differences underscores the complexity of auditory processing in psychosis. Moreover, ASSR measurements demonstrated moderate to high reliability over time, with persistent group differences despite symptom reduction. This stability suggests that ASSR could serve as a reliable biomarker for auditory processing deficits in psychosis. These findings support the notion that selective attention deficits are a fundamental aspect of psychosis, evident even at the level of basic auditory neurophysiology. The early identification of these deficits could enable targeted treatments for individuals at the highest risk of developing a chronic disorder.

## Figures and Tables

**Table 1:** Participant Demographics and IQ. Descriptive and inferential statistics are reported for first-episode schizophrenia subjects (FEP) and healthy controls (HC). Significant p-values are bolded. Values represent Mean ± SEM unless specified otherwise. M = male; F = female; W = White; B = Black or African American; A = Asian; N = American Indian or Alaska Native.

	FEP	HC	*t/χ* ^2^	*p-value*
Age at Baseline (years)	23.2 ± 4.0	25.1 ± 5.5	0.9	0.357
Sex (M/F)	24/16	24/16	1.0	0.999
Race (W/B/A/N)	16/19/4/1	24/5/9/2	12.1	**0.007**
Participant SES	30.0 ± 13.1	41.5 ± 14.5	3.7	**<0.001**
Parental SES	44.8 ± 13.5	49.3 ± 11.8	1.2	0.234
Education (years)	13.3 ± 2.0	15.9 ± 3.0	4.6	**<0.001**
WASI – IQ	101.1 ± 12.1	111.1 ± 8.4	3.7	**<0.001**
WASI – Verbal	49.7 ± 8.9	52.7 ± 6.3	1.5	0.141
WASI – Performance	51.2 ± 8.0	60.1 ± 5.6	6.1	**<0.001**

**Table 2:** Clinical and Cognitive Changes in First-Episode Schizophrenia (FEP). Descriptive statistics are reported for clinical and cognitive variables assessed at baseline and follow-up, as well as inferential statistics comparing these timepoints. Baseline measures are shown for all FEP (N = 40) as well as those FEP who were not lost to follow-up (N = 23). Statistics represent pairwise comparisons made between timepoints, within FEP not lost to follow-up. Significant p-values are bold. Values represent Mean ± SEM unless specified otherwise.

	Baseline (All FEP; N = 40)	Baseline (FEP w/Follow-up; N = 23)	Follow-up (N = 23)	*t*/X^2^	*p-value*
MATRICS Consensus Cognitive Battery					
Processing speed	36.7 ± 11.8	36.7 ± 11.0	38.2 ± 8.9	0.8	0.434
Attention	33.3 ± 10.7	35.0 ± 11.0	37.7 ± 10.6	1.4	0.164
Working memory	37.4 ± 11.6	35.8 ± 11.4	39.7 ± 9.7	2.0	0.053
Verbal learning	41.1 ± 9.7	40.1 ± 10.7	40.0 ± 7.5	0.4	0.700
Visual learning	37.9 ± 12.3	38.7 ± 14.2	37.6 ± 14.9	0.5	0.570
Reasoning	42.0 ± 10.9	43.4 ± 11.3	46.1 ± 12.5	1.1	0.269
Social cognition	44.6 ± 12.2	42.3 ± 14.0	42.9 ± 15.0	0.3	0.794
Total	32.3 ± 13.3	32.1 ± 15.0	34.3 ± 13.0	1.2	0.239
Symptoms					
PANSS – General	38.9 ± 8.8	38.1 ± 9.7	32.0 ± 9.2	3.1	**0.005**
PANSS – Negative	17.3 ± 6.0	17.0 ± 6.5	15.0 ± 5.6	2.1	**0.048**
PANSS – Positive	20.4 ± 6.2	20.2 ± 6.4	14.6 ± 6.5	3.9	**<0.001**
PANSS – Total	76.6 ± 17.6	75.4 ± 20.1	61.7 ± 18.9	3.6	**0.002**
PSYRATS - Hallucinations	11.7 ± 14.1	13.7 ± 14.5	6.2 ± 10.3	3.1	**0.005**
PSYRATS - Delusions	12.2 ± 7.2	11.2 ± 7.3	5.3 ± 6.0	3.9	**<0.001**
Auditory Hallucinations ±	18/22	11/12	7/16	1.5	0.227
Medication data					
Cpz. equivalent dose (mg)^a^					
Current	293 ± 234	255 ± 205	312 ± 245		
Lifetime (estimated)	5304 ± 4950	5642 ± 6127	85,374 ± 68,383		
Medicated^b^/unmedicated	29/11	15/8^c^	15/8^c^		

a Chlorpromazine (Cpz) equivalent dose is calculated only for medicated participants.

b Of the thirteen medicated participants at baseline, eight were prescribed risperidone, two were prescribed haloperidol, two were prescribed aripiprazole, one was prescribed paliperidone, and one was prescribed quetiapine (1 participant was prescribed both risperidone and haloperidol). Of the sixteen medicated participants at follow-up, seven were prescribed risperidone, six were prescribed aripiprazole, two were prescribed olanzapine, two were prescribed paliperidone, one was prescribed lurasidone, and one was prescribed haloperidol (3 participants were prescribed with two medications: risperidone/aripiprazole, haloperidol/olanzapine, and risperidone/paliperidone).

c Three unmedicated participants at follow-up were also unmedicated at baseline, while 5 had recently discontinued medication.

**Table 3:** Demographic, Clinical, and Cognitive variables in Healthy Controls (HC) and First-Episode Schizophrenia (FEP) with (AH+) and without (AH−) auditory hallucinations Descriptive statistics are reported for variables assessed at baseline, as well as inferential statistics comparing AH + vs AH−. Significant p-values are bold. Values represent Mean ± SEM unless specified otherwise.

	HC (N = 40)	AH− (N = 22)	AH+ (N = 18)	*t*/X^2^	*p-value*
Age at Baseline (years)	25.1 ± 5.5	23.0 ± 4.2	23.4 ± 3.9	−0.7	0.332
Sex (M/F)	24/16	15/7	12/5	1.0	0.999
Race (W/B/A/N)	24/5/9/2	9/10/2/1	7/9/2/0	0.3	0.968
Participant SES	41.5 ± 14.5	29.7 ± 15.2	30.4 ± 10.5	−0.2	0.853
Parental SES	49.3 ± 11.8	43.4 ± 13.8	46.6 ± 13.2	−0.7	0.464
Education (years)	15.9 ± 3.0	13.4 ± 2.4	13.2 ± 1.4	0.3	0.750
WASI – IQ	111.1 ± 8.4	98.7 ± 11.0	104.0 ± 13.0	−1.4	0.177
WASI – Verbal	52.7 ± 6.3	47.8 ± 8.0	52.1 ± 9.6	−1.5	0.135
WASI – Performance	60.1 ± 5.6	50.3 ± 8.7	52.3 ± 7.2	−0.8	0.416
MATRICS Consensus Cognitive Battery					
Processing speed	52.7 ± 8.9	35.7 ± 12.8	37.8 ± 10.7	−0.6	0.574
Attention	49.0 ± 9.5	31.9 ± 10.6	34.9 ± 11.1	−0.9	0.394
Working memory	49.9 ± 9.4	35.9 ± 10.5	39.4 ± 12.9	−0.9	0.349
Verbal learning	55.2 ± 9.3	41.1 ± 8.1	42.8 ± 11.6	−0.5	0.605
Visual learning	46.1 ± 8.3	37.2 ± 12.7	38.8 ± 12.0	−0.4	0.676
Reasoning	50.5 ± 8.8	39.4 ± 8.7	45.2 ± 12.7	−1.6	0.113
Social cognition	52.0 ± 8.7	44.0 ± 12.2	45.4 ± 12.5	−0.4	0.718
Total	51.0 ± 8.2	30.4 ± 12.7	34.7 ± 14.0	−1.0	0.315
Symptoms					
PANSS – General		36.3 ± 9.6	42.2 ± 6.7	−2.3	**0.030**
PANSS – Negative		17.1 ± 6.3	17.4 ± 5.9	−0.1	0.896
PANSS – Positive		17.6 ± 6.1	23.7 ± 4.5	−3.6	**0.001**
PANSS – Total		71.0 ± 18.4	83.3 ± 14.2	−2.4	**0.023**
PSYRATS - Hallucinations		0 ± 0	25.9 ± 8.5		
PSYRATS - Delusions		9.8 ± 8.3	14.9 ± 4.4	−2.4	**0.019**
Medication data					
Cpz. equivalent dose (mg)^a^					
Current		343 ± 271	241 ± 181	1.2	0.240
Lifetime (estimated)		7843 ± 9389	3049 ± 3989	2.1	**0.038**
Medicated**/unmedicated		15/7	14/4		

a Chlorpromazine (Cpz) equivalent dose is calculated only for medicated participants.

**Table 4:** Sequential Multiple Regression of (1) Auditory Hallucination Presence/Absence (AH±), (2) left A1 ASSR (passive), and (3) right A1 ASSR (active) on Auditory Hallucination Severity measured with PSYRATS in FEP (N = 40).

	B	SE B	β	r_partial_	R^2^	ΔR^2^
**Step 1**: AH±	25.89	1.80	0.92	0.92*	0.85*	
**Step 2**: AH±	23.85	2.50	0.85	0.91*	0.88*	0.03*
Left A1 ASSR (Passive)	2.33	0.76	0.19	0.45*		
**Step 3**: AH±	22.88	1.61	0.81	0.92*	0.90*	0.03*
Left A1 ASSR (Passive)	2.72	0.69	0.22	0.54*		
Right A1 ASSR (Active)	−1.72	0.55	−0.17	−0.46*		

Asterisks represent statistical significance (*p* < 0.05). Asterisks are not shown for beta statistics, as *p*-values for these statistics are identical to partial correlation statistics.

**Table 5:** Sequential Multiple Regression of left A1 ASSR (passive) and right A1 ASSR (active) on Auditory Hallucination Severity measured with PSYRATS. Regression models include only those FEP who report auditory hallucinations (N = 18).

	B	SE B	β	r_partial_	R^2^	ΔR^2^
**Step 1**: Left A1 ASSR (Passive)	3.40	1.31	0.54	0.54*	0.30*	
**Step 2**: Left A1 ASSR (Passive)	3.59	1.01	0.57	0.65*	0.55*	0.25*
Right A1 ASSR (Active)	−3.00	1.00	−0.50	−0.60*		

Asterisks represent statistical significance (*p <* 0.05). Asterisks are not shown for beta statistics, as *p*-values for these statistics are identical to partial correlation statistics.
